# The Role of Bloom Index of Gelatin on the Interaction with Retinal Pigment Epithelial Cells

**DOI:** 10.3390/ijms10083442

**Published:** 2009-08-03

**Authors:** Jui-Yang Lai

**Affiliations:** 1 Institute of Biochemical and Biomedical Engineering, Chang Gung University, Taoyuan 33302, Taiwan; 2 Biomedical Engineering Research Center, Chang Gung University, Taoyuan 33302, Taiwan; 3 Molecular Medicine Research Center, Chang Gung University, Taoyuan 33302, Taiwan

**Keywords:** gelatin, Bloom index, *in vitro* biocompatibility, retinal pigment epithelial cells

## Abstract

Biocompatible materials are of considerable interest in the development of cell/drug delivery carriers for therapeutic applications. This paper investigates the effects of the Bloom index of gelatin on its interaction with retinal pigment epithelial (RPE) cells. Following two days of culture of ARPE-19 cells with gelatin samples G75-100, G175, and G300, the *in vitro* biocompatibility was determined by cell proliferation and viability assays, and glutamate uptake measurements, as well as cytokine expression analyses. The mitochondrial dehydrogenase activity in the G300 groups was significantly lower than that of G75-100 and G175 groups. The Live/Dead assays also showed that the gelatin samples G300 induced mild cytotoxicity. In comparison with the treatment of gelatins with low Bloom index, the exposure to high Bloom strength gelatins markedly reduced the glutamate uptake capacity of ARPE-19 cells. One possible explanation for these observations is that the presence of gelatin samples G300 with high viscosity in the medium may affect the nutrient availability to cultured cells. The analyses of pro-inflammatory cytokine IL-6 expression at both mRNA and protein levels showed that the gelatins with low Bloom index caused less cellular inflammatory reaction and had more acceptable biocompatibility than their high Bloom strength counterparts. These findings suggest that the Bloom index gives influence on cellular responses to gelatin materials.

## 1. Introduction

Gelatin is a naturally occurring biopolymer derived from collagen, which is the major structural protein in the connective tissue of animal bone and skin. In the food industry, gelatin has long been used as stabilizer, clarification agent, and protective coating material [1]. Because of its excellent bioaffinity, gelatin has also been extensively applied in the medical field: as a sealant for vascular prostheses, an adhesive and in absorbent pads for surgical use [2]. As a protein-based biomaterial, gelatin is biodegradable and has no antigenicity under physiological conditions. Thus, it is an indispensable ingredient in modern pharmaceuticals. Tabata *et al.* [3] have explored gelatin hydrogels as a delivery system to achieve the controlled release of biologically active growth factors. When electrostatically complexed with a positively or negatively charged gelatin, an oppositely charged protein can be modified to increase its stability, targeting, and sustained release, leading to enhanced therapeutic efficacy. Up to now, various gelatin-based dosage forms including capsules [4], microspheres [5], and nanoparticles [6], have already been proposed as promising carriers for drug delivery.

The bioadhesive nature of gelatin offers another advantage, that of being able to deliver cell transplants for tissue repair. We recently reported the successful fabrication of bioengineered human corneal endothelial cell sheets from thermo-responsive poly(*N*-isopropylacrylamide) (PNIPAAm)-grafted culture surfaces [7]. To overcome the fragility of sheet grafts, gelatin hydrogel discs have been used as supporting materials for transportation and surgical handling [8]. In a model of corneal endothelial regeneration in rabbits, it has been shown that the physicochemical characteristics (*i.e.,* isoelectric point and molecular weight) of gelatin play a very prominent role in the functionality of cell sheet delivery carriers and in the therapeutic effect of gelatin-cell sheet constructs [9–11]. Structurally, gelatin molecules consist of repeating sequences of glycine-X-Y triplets, in which X and Y are often proline and hydroxyproline amino acid residues [12]. These sequences have been related to the ability of the gelatin to form thermally reversible gels. Our previous work has demonstrated that the gelatin exhibiting thermo-responsive sol-gel transition behavior has potential use in retinal regenerative medicine [13,14]. By means of thermal-conductive trephination, two overlapping gelatin membranes were sealed circularly to encapsulate intact fetal retinas for transplantation.

It is known that the material biocompatibility is a crucial factor in determining the success of new medical implants and devices in the body. Despite having several attractive features, gelatin materials need to be tested to check their biocompatibility before their use as carriers for intraocular delivery of cell/tissue sheets. Gelatin exhibits very high bioabsorptivity when placed *in vivo*. A study from Guidoin *et al.* [15] suggested that the implanted gelatin matrices were absorbed in the canine thoracic aorta after between 7 and 14 days. Their study seems to show that the use of a bioerodible gelatin coating can promote cellular regeneration and achieve complete biological healing. More recently, we have demonstrated that the transplantation of gelatin hydrogels into the anterior chamber of the rabbit eyes causes significant corneal edema and elevated intraocular pressure in the early postoperative phase (within 3 days) [16]. The tissue responses may resolve rapidly with *in vivo* biodegradation of gelatin in an ocular immune privileged site. Furthermore, the absorption of gelatin membranes sterilized with 16.6 kGy gamma irradiation occurred within a few days after implantation in the subretinal space of rabbits [13,14]. The eyes receiving low Bloom strength gelatin carriers had approximately 7–9 rows of photoreceptor nuclei in the outer nuclear layer of the retina, indicating good biocompatibility *in vivo*. In comparison, high Bloom strength gelatin samples have been associated with significant inflammatory infiltration in the retinal tissues.

In general, biocompatibility is governed mainly by the interface between foreign materials and host living cells/tissues. The intrinsic nature of the material including chemical composition, molecular weight, charge, and hydrophilicity may have an impact on the biocompatibility. Gelatin is obtained by the thermal, chemical, or physical denaturation of collagen. The treatment of lower extraction temperature can produce gelatin with greater Bloom index, which is a measure of the stiffness of gelatin gels [17]. It has been reported that the increase in Bloom index of the gelatin leads to a remarkable improvement in the mechanical properties and a significant reduction in water-absorbing capacity of the membranes [14,18]. In addition, the Bloom strength strongly affects the atomistic structure of gelatin [17] and therefore its biomedical applications including tissue engineering [14] and drug delivery [19]. Since gelatin is an attractive biopolymer for ophthalmic use, this work aims to analyze the *in vitro* material biocompatibility using human retinal pigment epithelial (RPE) cell line cultures. The cell proliferation, viability, and glutamate uptake, as well as pro-inflammatory cytokine expression were studied to give insight into the Bloom index effects on cellular responses to gelatin.

## 2. Results and Discussion

### 2.1. Rheological Measurements

**Figure 1.**
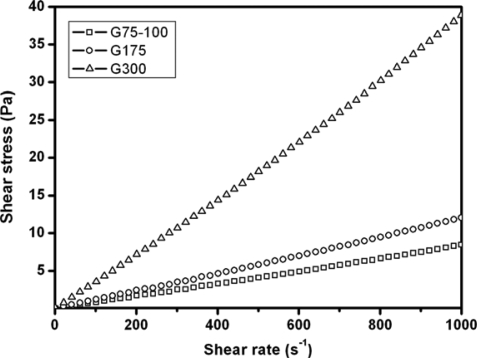
Shear stress versus shear rate curves for various gelatin samples in BSS at 37 °C.

Typical rheograms for gelatin samples G75-100, G175, and G300 in BSS at 37 °C are shown in Figure 1. The flow curves of various gelatin solutions exhibit a linear relationship over the entire rate range. The viscosity of the fluid is defined as the ratio of the shear stress to the shear rate. In this study, all three types of gelatins showed a Newtonian fluid behavior because the viscosities are independent of the shear rate. Furthermore, the viscosity was increased with increasing the Bloom index of gelatin.

It is known that the triple-helical regions of collagen are stabilized by hydrogen bonding and van der Waals attractions between imino residues on different chains [20]. Conversion of collagen to gelatin involves disruption of these noncovalent interactions and leads to a decrease in molecular order. Bigi *et al.* [18] have shown that the triple-helix content of gelatin increased with its Bloom number. In addition to the molecular weight, the chemical structure can affect the resultant viscosity of gelatin. A recent study from Peng *et al.* [21] suggested that the increase in viscosity was related to the chain organization of gelatin in solution, *e.g.,* the formation of a helix structure. Our present results were compatible with their findings and indicated that high Bloom strength gelatins have higher viscosity than the samples with low Bloom index. The material degradation occurred rapidly when the gelatin was implanted in the anterior chamber [16] or the subretinal space [13,14]. Since gelatin is viscous, its degradation products residing in an ocular immune-privileged site may contribute to the increase in viscosity of tissue fluids. It should be investigated whether this effect is detrimental to living cells and tissues. Here, we performed an *in vitro* study to examine the role of Bloom index on the biocompatibility of gelatin.

### 2.2. Cell Proliferation Assays

**Figure 2.**
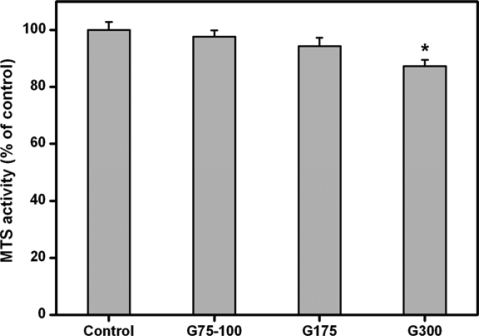
Cell proliferation assay of ARPE-19 cell cultures incubated in the presence of various dissolved gelatin materials for two days at 37 °C. Results are expressed as percentage of controls (MTS activity of cells cultured in the absence of materials). An asterisk indicates statistically significant differences (*p<0.05; *n* = 4) as compared to controls.

Figure 2 shows the results of quantitative analysis for ARPE-19 cell growth. After two days of culture, similar levels of mitochondrial dehydrogenase activity (MTS activity) were observed in the control, G75-100, and G175 groups and not statistically different (*p*>0.05). By contrast, in the G300 groups, the MTS activity was significantly reduced by about 13% (*p*<0.05) as compared to that of the control groups. In addition, the cells exposed to gelatins with higher Bloom strengths showed lower density. These results indicate that the Bloom index of gelatin may play a crucial role in the regulation of ARPE-19 cell proliferation.

The interaction between cells and biopolymers is the evaluation indicator of cell compatibility of materials [22]. RPE is composed of a monolayer of cuboidal cells separated from the neural retina by the subretinal space. The RPE cells are known to mediate the transport of metabolic intermediates, waste products, ions, and fluid components between the choriocapillaries and the retina [23]. As a protein-based biomaterial, gelatin is substantially nontoxic. We have previously shown that the high Bloom strength gelatins sterilized with gamma irradiation seem to be cytostatic toward rat iris pigment epithelial cells [14]. In this study, similar results were obtained from gelatin samples sterilized by ethanol treatment. Our findings suggest that the higher Bloom index of gelatins is related to the inhibitory effects of ARPE-19 cell growth. One possible explanation for these observations is that the presence of high Bloom strength gelatins in the culture system leads to an increase in the medium viscosity and to problem of mass transfer. The insufficient nutrient availability to the ARPE-19 cells may limit their proliferation and metabolic responses. Therefore, the gelatins with lower Bloom index are more suitable for use as carrier materials for ocular tissue engineering and drug delivery.

### 2.3. Cell Viability Assays

**Figure 3.**
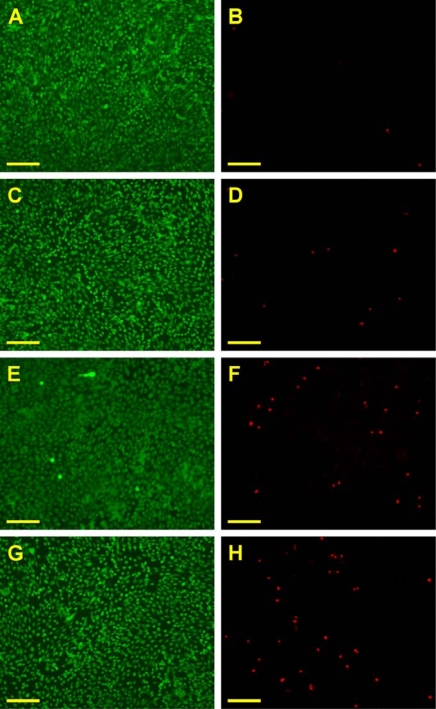
Cell viability of ARPE-19 cultures was determined by staining with Live/Dead Viability/Cytotoxicity Kit in which the live cells fluoresce green and the dead cells fluoresce red. Green (A, C, E, G) and red (B, D, F, H) fluorescence images of cells in (A, B) controls (without materials) after exposure to various dissolved gelatin materials (C, D) G75-100, (E, F) G175, and (G, H) G300 for 2 days at 37 °C. Scale bars indicate 100 μm.

**Figure 4.**
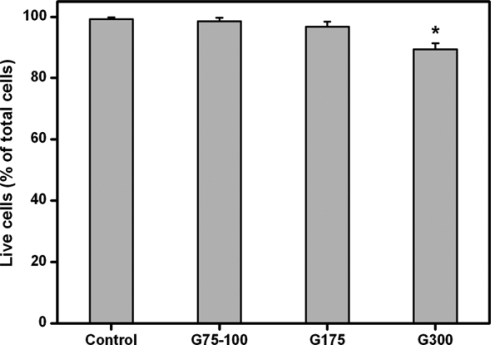
Mean percentage of live cells in the ARPE-19 cultures exposed to various dissolved gelatin materials as measured by the Live/Dead assay. An asterisk indicates statistically significant differences (*p<0.05; *n* = 6) as compared to controls (without materials).

Figure 3 is a representative photograph of ARPE-19 cells labeled with Live/Dead stain, where the live cells fluoresce green and the dead cells fluoresce red. In the control groups, the majority of confluent cell cultures are viable (Figure 3A and B). After exposure to gelatin samples G75-100 for two days at 37 °C, the ARPE-19 cultures maintain good viability with only a few dead cells (Figure 3C and D). By contrast, in the G175 and G300 groups, an increased number of red-stained nuclei were noted, indicating that these gelatin samples induced mild cytotoxicity (Figure 3E-H). Figure 4 shows the mean percentage of live cells as determined by the Live/Dead assay. The cell viability was significantly lower in the G300 groups (89.4 ± 1.9%), compared with those of the control (99.2 ± 0.6%), G75-100 (98.5 ± 1.2%), and G175 groups (96.7 ± 1.7%; *p*<0.05). These findings suggest that the gelatins with low Bloom index are more cytocompatible than their high Bloom strength counterparts.

Because of its *in vivo* compatibility and safety, gamma-ray-sterilized gelatin has been used as a matrix for harvested RPE [24] and retinal sheets [13,25]. Using a Live/Dead assay kit, the cell viability of RPE sheets exposed to gelatin (Bloom index = 300) was assessed and was found to be more than 85% 24 h from harvesting [24]. Similar to their findings, our results indicate that the viability of RPE cell line cultures is approximately 90% after incubation with high Bloom strength gelatin samples for two days. However, in this study, we have noted the effects of Bloom index of gelatin on cell compatibility of materials *in vitro*. The negligible cytotoxicity of the gelatins with low Bloom index makes them promising candidates for ophthalmic applications.

### 2.4. Glutamate Uptake Measurements

**Figure 5.**
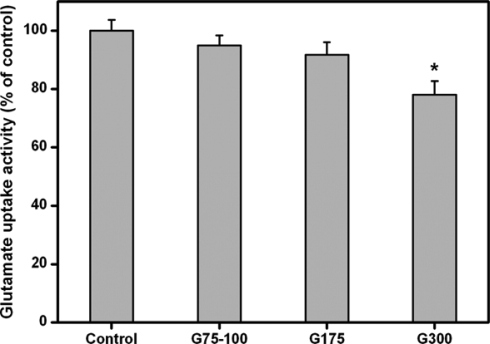
Glutamate uptake in the ARPE-19 cultures exposed to various dissolved gelatin materials for 2 days. Data in the experimental groups are percentages relative to that of control groups (without materials). An asterisk indicates statistically significant differences (*p<0.05; *n* = 3) as compared to controls.

Figure 5 shows the results of glutamate uptake of ARPE-19 cells. After 2 days in culture, the glutamate uptake activity did not show a significant difference between the control, G75-100, and G175 groups (*p*>0.05). However, there was a significant reduction of activity (>20% reduction) in the G300 groups as compared to that of the control groups (*p*<0.05). These results clearly demonstrate that the Bloom index of gelatin may be a potential factor involved in the regulation of glutamate uptake in RPE cells exposed to foreign materials.

In the present study, the ARPE-19 cells were cultured with gelatins in the medium for *in vitro* glutamate uptake assays. We have previously shown that gelatin has a high degree of water binding capability and can absorb a tremendous amount of water at up to 10 times its weight [10]. The mechanism of hydration of gelatin is a capillary phenomenon of water molecules penetrating the tiny interstices of a collagen-like four-dimensional structure in the gelatin [26]. During this process, the medium nutrients may be simultaneously entrapped in the gelatin materials. Glutamate is the main excitatory neurotransmitter in the retina but is neurotoxic when present in excessive amounts [27]. Therefore, the glutamate concentration in the subretinal space should be maintained at a fairly constant level. The RPE is responsible for the turnover and renewal of the photoreceptor and participates in regulating the glutamate uptake [28]. The results of this study showed that the exposure to high Bloom strength gelatins markedly reduced the glutamate uptake capacity of ARPE-19 cells. This is probably due to that the presence of gelatin with high viscosity in the culture medium may affect the nutrient availability to RPE cells.

### 2.5. Cytokine Expression Analyses

**Figure 6.**
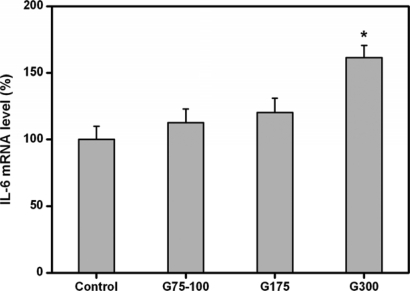
Gene expression of IL-6 in ARPE-19 cells incubated with various dissolved gelatin materials for two days by real-time RT-PCR. Normalization was done by using GAPDH. Data in the experimental groups are percentages relative to that of control groups (without materials). An asterisk indicates statistically significant differences (*p<0.05; *n* = 3) as compared to controls.

It has been reported that elevated IL-6 level is associated with severe foreign body reaction to the implanted materials [29]. A study from Abe *et al.* [30] suggested that the IL-6 was involved in the local reaction of retinal tissues after the early days of transplantation of human RPE. In this study, we therefore analyzed the expression of IL-6 in RPE cell cultures for indication of *in vitro* biocompatibility. Figure 6 shows the pro-inflammatory gene expression of ARPE-19 cells exposed to various gelatin membranes for two days. Similar IL-6 gene expression levels were observed in the control, G75-100, and G175 groups and not statistically different (*p*>0.05). By contrast, the expressions of these genes in the G300 groups were significantly higher than those of the other groups (*p*<0.05), indicating that the IL-6 genes are up-regulated after culture with high Bloom strength gelatins.

**Figure 7.**
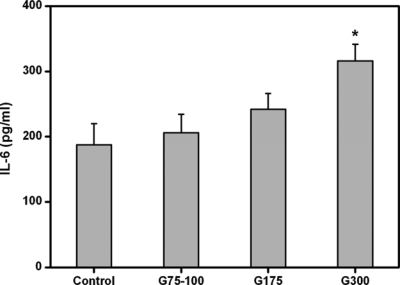
Level of IL-6 released from ARPE-19 cell cultures after incubation with various dissolved gelatin materials for two days. An asterisk indicates statistically significant differences (*p<0.05; *n* = 4) as compared to controls (without materials).

The ARPE-19 cell secretion of IL-6 in response to various gelatin membranes is also shown in Figure 7. The expression of IL-6 in the control, G75-100, and G175 groups was 187.4 ± 32.5, 206.1 ± 28.2, and 241.6 ± 24.3 pg/mL, respectively. The values did not show a significant difference between these three groups (*p*>0.05), which indicates that the exposure to gelatins with low Bloom index does not promote inflammation. However, the IL-6 level was significantly higher in the G300 groups (315.8 ± 26.1 pg/mL), compared with those of the low Bloom strength gelatin groups (*p*<0.05). These findings suggest that the Bloom index of gelatin has an important influence on the stimulation of pro-inflammatory cytokine IL-6 production in RPE cells.

Recently, we reported that the exposure of human corneal endothelial cells to the gelatins of higher molecular weight induces higher levels of IL-6 [10]. In addition to material chemistry, the material morphology has been suspected to be responsible for pro-inflammatory cytokine production. Reichert *et al.* [31] have investigated the temporal cytokine expression profile from human THP-1 monocytes exposed to phagocytosable Ti particles and to Ti discs of comparable surface roughness and concluded that cells treated by disc samples may produce in many instances a higher cytokine expression than did particles. In this study, cytokine expression analyses for the cell cultures were performed in order to further compare the effects of Bloom index of gelatin. The expression at both mRNA and protein levels were characterized using the techniques of quantitative real-time RT-PCR and ELISA. In comparison with high Bloom strength samples, the gelatins with low Bloom index may cause less cellular inflammatory reaction and have more acceptable biocompatibility.

## 3. Experimental Section

### 3.1. Materials

Gelatins, prepared through an acid process treatment of pig skin, were purchased from Sigma-Aldrich (St. Louis, MO, USA). The Bloom index indicates the gel strength of the gelatin, defined as the weight in grams necessary to apply to the surface of gelatin gel, to produce a 4 mm depth depression [14]. In the present study, the gelatins had different Bloom numbers (75–100, 175, and 300), with the higher value producing stronger gels. A gelatin sample with a Bloom number of 75–100 was designated as G75-100. Balanced salt solution (BSS, pH 7.4) was obtained from Alcon Laboratories (Fort Worth, TX, USA). Phosphate-buffered saline (PBS, pH 7.4) was purchased from Biochrom AG (Berlin, Germany). Dulbecco’s modified Eagle’s medium/Ham’s F12 nutrient mixture (DMEM/F12) and TRIzol reagent were purchased from Gibco-BRL (Grand Island, NY, USA). Fetal bovine serum (FBS) and the antibiotic/antimycotic (A/A) solution (10,000 U/mL penicillin, 10 mg/mL streptomycin and 25 μg/mL amphotericin B) were obtained from Biological Industries (Kibbutz Beit Haemek, Israel). Radioactive [^3^H]glutamate was purchased from Amersham (Little Chalfont, UK). All the other chemicals were of reagent grade and used as received without further purification.

### 3.2. Preparation of Gelatin Membranes

The gelatin membranes were prepared by solution casting methods as described elsewhere [13,14]. Briefly, an aqueous solution of 10 wt% gelatin was cast into a polystyrene planar mold (5 × 5 cm^2^, 1.5 cm depth), and air-dried for 24 h at 25 °C to obtain membranes (about 30 μm thick). Then, the membrane samples were sterilized in a 70% ethanol solution overnight.

### 3.3. Rheological Measurements

To measure the rheological behavior of gelatin, the membrane samples (1 mg) were dissolved in 1 mL of physiological medium (BSS) at 37 °C. The shear stress versus shear rate curves and flow properties of resulting gelatin solutions were determined at 37 °C in a rheometer (Carri-Med CSL2100; TA Instruments, Newcastle, UK). A double concentric cylinder system and a cone plate sensor with a 2° cone angle and 6 cm in diameter were used for evaluating flow properties. The shear rate was altered in the range from 0.1 to 1000 s^−1^ during rheological measurements.

### 3.4. Human RPE Cell Line Cultures

ARPE-19 cells, a spontaneously immortalized human cell line (BCRC No. 60383) with morphological and functional characteristics similar to adult human RPE [32], were purchased from the Bioresource Collection and Research Center (Hsinchu, Taiwan, ROC). The cells were maintained in regular growth medium containing DMEM/F12, 10% FBS, and 1% A/A solution. Cultures were incubated in a humidified atmosphere of 5% CO_2_ at 37 °C. The cells from passage 32 were used for experiments.

### 3.5. In Vitro Biocompatibility Tests

The sterilized gelatin membranes were thoroughly rinsed in PBS and completely dissolved in fresh culture medium at 37 °C. After 48 h of incubation with medium containing gelatin materials, the cultures were examined by various *in vitro* tests. The gelatin was presented as a soluble protein on the cell layers during biocompatibility testing. ARPE-19 cells in regular growth medium without gelatins served as control groups.

#### 3.5.1. Cell Proliferation Assays

ARPE-19 cells (7 × 10^4^ cells/well) were seeded in 24-well plates containing 1 mL of regular growth medium and incubated overnight to allow cell attachment. Then, the medium was replaced with culture medium containing gelatin materials. After incubation at 37 °C for 2 days, cell growth was estimated using the CellTiter 96 Aqueous Non-Radioactive Cell Proliferation MTS Assay (Promega, Madison, WI, USA), in which MTS tetrazolium compound is bio-reduced by cells to form a water-soluble colored formazan. The amount of colored product is proportional to the number of metabolically active cells. 100 μl of the combined MTS/PMS (20:1) reagent was added to each well of the 24-well plate, and incubated for 3 h at 37 °C in a CO_2_ incubator. The data of absorbance readings at 490 nm were measured using the Multiskan Spectrum Microplate Spectrophotometer (ThermoLabsystems, Vantaa, Finland). All experiments were performed in quadruplicate, and the results were expressed as relative MTS activity when compared to control groups.

#### 3.5.2. Cell Viability Assays

ARPE-19 cells were seeded in 24-well plates containing regular growth medium and allowed to grow to confluency. The medium was subsequently replaced with culture medium containing gelatin materials. After incubation at 37 °C for 2 days, cell viability was determined using a membrane integrity assay, the Live/Dead Viability/Cytotoxicity Kit (Molecular Probes, Eugene, OR, USA) which contains calcein AM and ethidium homodimer-1 (EthD-1). It depends on the intracellular esterase activity to identify the living cells, which cleaves the calcein AM to produce a green fluorescence. In dead cells, EthD-1 can easily pass through the damaged cell membranes to bind to the nucleic acids, yielding a red fluorescence. After washing three times with PBS, the cultures were stained with a working solution consisting of 2 μL of EthD-1, 1 mL of PBS, and 0.5 μL of calcein AM. Under fluorescence microscopy (Axiovert 200M; Carl Zeiss, Oberkochen, Germany), three different areas each containing approximately 500 cells were counted at 100× magnification. All experiments were performed in duplicate, and the viability of the ARPE-19 cell cultures was expressed as the average ratio of live cells to the total number of cells in these six different areas.

#### 3.5.3. Glutamate Uptake Measurements

For *in vitro* glutamate uptake assays, ARPE-19 cells (3 × 10^4^/cm^2^) were seeded in 35-mm tissue culture dishes containing 2 mL of regular growth medium and allowed to grow to confluency. Before testing, the cells were washed three times with Krebs-Ringer-Hepes (KRH) buffer (126 mM NaCl, 5.1 mM KCl, 0.81 mM CaCl_2_, 1.3 mM MgSO_4_, 1.3 mM NaH_2_PO_4_, 15 mM Hepes and 10 mM d-glucose, pH 7.4). The medium was replaced with culture medium containing gelatin materials. After incubation at 37 °C for 2 days, the radioactive [^3^H]glutamate solution, which contained 1.25 μCi [^3^H]glutamate, and the total glutamate concentration of 5 μM were added to the dishes. After a further incubation for 10 min, the reaction was terminated by washing three times with ice-cold KRH buffer. The dried cells were dispersed with 0.4 N NaOH and neutralized with HCl. The radioactivity inside the cells was quantitated by using a liquid scintillation counter. The results were calculated to the protein content of the cells and expressed as specific activity relative to that of control groups. All experiments were conducted in triplicate.

The protein content of the cells was determined by colorimetric assay using a bicinchoninic acid (BCA) protein assay kit (Pierce Chemical, Rockford, IL, USA). The working solution was prepared by mixing a 4% copper (II) sulfate pentahydrate solution with an excess of BCA at a final ratio of 1:50 v/v; 100 μL of test samples was added to 2 mL of the working reagent. The mixture was incubated at 37 °C for 30 min, and was subsequently cooled to room temperature. The absorbance of the mixture solution was recorded with an UV-visible spectrophotometer (Thermo Scientific, Waltham, MA, USA) at 562 nm using bovine serum albumin at various known concentrations as standard.

#### 3.5.4. Cytokine Expression Analyses

ARPE-19 cells were grown to confluence on 24-well plates in regular growth medium. Then, the medium was replaced with culture medium containing gelatin materials. Following two days of culture, interleukin-6 (IL-6) expression was detected at both messenger RNA (mRNA) and protein levels.

Total RNA was isolated from ARPE-19 cells with TRIzol reagent according to the manufacturer’s procedure. Reverse transcription of the extracted RNA (1 μg) was performed using ImProm-II (Promega) and Oligo(dT)_15_ primers (Promega). The primers used to amplify the human IL-6 complementary DNA (cDNA) were 5′-CCACTCACCTCTTCAGAACGAA-3′ (sense) and 5′-GGCAAGTCTCCTCATTGAATCC-3′ (antisense), and those used to amplify the internal control cDNA, glyceraldehyde-3-phosphate dehydrogenase (GAPDH), were 5′-TGGTATCGTGGAAGGACT CATGAC-3′ (sense) and 5′-ATGCCAGTGAGCTTCCCGTTCAGC-3′ (antisense). Quantitative real-time reverse transcription polymerase chain reaction (RT-PCR) was performed on a Light-Cycler instrument (Roche Diagnostics, Indianapolis, IN, USA) according to the manufacturer’s instructions with FastStart DNA Master SYBR Green I reagent (Roche Diagnostics). Each sample was determined in triplicate and the results for IL-6 were normalized to the level of GAPDH mRNA.

Aliquots of the supernatant were collected to measure the IL-6 levels. The release of IL-6 from cultivated cells into the conditioned medium was detected by the Quantikine enzyme-linked immunosorbent assay (ELISA) kit (R&D Systems, Minneapolis, MN, USA) specific for human IL-6. Cytokine bioassays were performed according to the manufacturer’s instructions. Photometric readings at 450 nm were measured using the Spectrophotometer (ThermoLabsystems). Results were expressed as pg/ml. All experiments were conducted in quadruplicate.

### 3.6. Statistics

Results were expressed as mean ± standard deviation. Comparative studies of means were performed using one-way analysis of variance (ANOVA). Significance was accepted with *p*<0.05.

## 4. Conclusions

Biocompatible materials are of considerable interest in the development of cell/drug delivery carriers for therapeutic applications. This paper investigates the effects of Bloom index of gelatin on its interaction with RPE cells. The results of *in vitro* studies including cell proliferation and viability assays, and glutamate uptake measurements, as well as cytokine expression analyses showed that the low Bloom strength gelatins were more cytocompatible than their high Bloom strength counterparts. It is concluded that the Bloom index gives influence on cellular responses to gelatin materials.
